# Host cell maturation modulates parasite invasion and sexual differentiation in *Plasmodium berghei*

**DOI:** 10.1126/sciadv.abm7348

**Published:** 2022-04-27

**Authors:** Franziska Hentzschel, Matthew P. Gibbins, Charalampos Attipa, Dario Beraldi, Christopher A. Moxon, Thomas D. Otto, Matthias Marti

**Affiliations:** 1 Wellcome Centre for Integrative Parasitology, University of Glasgow, Glasgow, UK.; 2 Department of Tropical Disease Biology, Liverpool School of Tropical Medicine, Liverpool, UK.; 3 Department of Pathology, Kamuzu University of Health Sciences, Blantyre, Malawi.; 4 Malawi-Liverpool-Wellcome Clinical Research Programme, Kamuzu University of Health Sciences, Blantyre, Malawi.; 5 Department of Paediatrics and Child Health, Kamuzu University of Health Sciences, Blantyre, Malawi.; 6 Department of Immunology and Infectious Diseases, Harvard School of Public Health, Boston, MA, USA.

## Abstract

Malaria remains a global health problem causing more than 400,000 deaths annually. *Plasmodium* parasites, the causative agents of malaria, replicate asexually in red blood cells (RBCs) of their vertebrate host, while a subset differentiates into sexual stages (gametocytes) for mosquito transmission. Parasite replication and gametocyte maturation in the erythropoietic niches of the bone marrow and spleen contribute to pathogenesis and drive transmission, but the mechanisms underlying this organ enrichment remain unknown. Here, we performed a comprehensive analysis of rodent *P. berghei* infection by flow cytometry and single-cell RNA sequencing. We identified CD71 as a host receptor for reticulocyte invasion and found that parasites metabolically adapt to the host cell environment. Transcriptional analysis and functional assays further revealed a nutrient-dependent tropism for gametocyte formation in reticulocytes. Together, we provide a thorough characterization of host-parasite interactions in erythropoietic niches and define host cell maturation state as the key driver of parasite adaptation.

## Teaser

Single-cell analysis of *Plasmodium* infection identifies host cell maturation as key driver of malaria parasite adaptation.

## INTRODUCTION

Malaria is caused by apicomplexan parasites of the genus *Plasmodium*. Despite continuous elimination efforts, the disease remains a global health problem with more than 400,000 deaths annually (*1*). In the vertebrate host, *Plasmodium* parasites replicate asexually in red blood cells (RBCs), and this asexual erythrocytic cycle causes the symptoms of the disease. A subset of erythrocytic parasite stages switches to sexual forms, the gametocytes. Sexual stages are the only form that is infectious to the mosquito and hence essential for transmission; however, the signals triggering stage conversion are not fully understood. *Plasmodium* development in the blood is tightly linked to erythropoiesis, the generation of new RBCs in the extravascular space of erythropoietic organs (*2*).

**Fig. 1.**
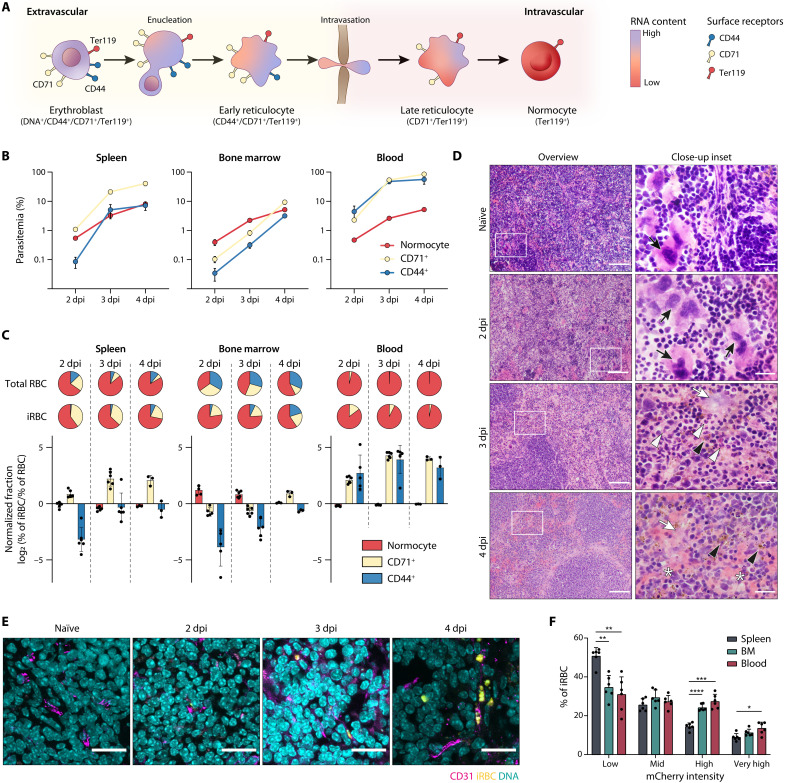
*P. berghei* host cell preference in the spleen, bone marrow, and blood. (**A**) Schematic depiction of erythropoiesis, including surface receptors indicative of erythroblasts, extravascular and intravascular reticulocytes, and normocytes. (**B**) Parasitemia per host organ and cell after infection with 1 × 10^6^ iRBC i.v. (intravenously) (*n* = 6 except for 4 dpi, where *n* = 3). (**C**) Pie charts: Average cell composition of RBCs (top row) and iRBCs (bottom row) per day and organ. Bar plots: Normalized fraction of host cell types in iRBC versus RBCs per organ. Values > 0 indicate preferential invasion of this host cell type, and values < 0 indicate decreased invasion. Same groups as in (B). (**D**) Representative histology images of naïve and infected spleen. Left column: Scale bars, 100 μm. Box indicates area of close-up. Right column: Scale bars, 20 μm. Black arrows, megakaryocytes indicative of hematopoiesis; white arrows, necrosis; black arrowheads, pigmented macrophages; white arrowheads, neutrophils; asterisks, hemorrhage. (**E**) Representative immunofluorescence images of spleen sections stained for CD31 (magenta). Parasites are mCherry-fluorescent (pseudo-colored yellow). Scale bars, 20 μm. (**F**) Percentage of parasites per mCherry gate per organ as proxy for parasite stages 3 dpi (*n* = 6, two-way ANOVA and Tukey’s post test). (B, C, and F) Means ± SEM. *P < 0.05; **P < 0.01; ***P < 0.001; ****P < 0.0001.

During erythropoiesis, hematopoietic stem cells differentiate into erythroblast precursors, which enucleate to give rise to reticulocytes. These reticulocytes then undergo further maturation in the extravascular parenchyma before intravasation into the circulation to complete their development to biconcave normocytes. Reticulocyte maturation is accompanied by organelle clearance, loss of RNA content, volume reduction, and surface remodeling (*3*–*6*). In mice, remodeling includes the loss of CD44 surface expression upon intravasation of the reticulocyte and the shedding of the transferrin receptor CD71 during the final maturation in circulation (Fig. 1A) (*6*, *7*). In humans, CD44 surface expression is maintained longer, yet other aspects of terminal erythropoiesis are thought to be similar to the murine model (*4*, *6*, *8*, *9*). The bone marrow is the main site for steady-state erythropoiesis in rodents and humans. In rodents, the spleen is also an erythropoietic organ and contributes substantially to overall RBC production, in particular, during stress erythropoiesis and anemia (*8*, *10*). In addition, the spleen is thought to play a role in the final maturation and CD71 shedding of circulating reticulocytes both in mice and humans (*11*–*14*).

Blood stage infection in the erythropoietic niche is an emerging paradigm in malaria parasites (*2*). The reticulocyte is a metabolically active and nutrient-rich host cell (*6*, *11*, *15*). It is thus not unexpected that many *Plasmodium* species, including the human malaria parasite *P. falciparum* and the rodent parasite *P. berghei*, preferentially invade younger RBCs. Some species, including the human malaria parasite *P. vivax*, are restricted to reticulocytes (*12*–*14*, *16*, *17*). In addition, extravascular maturation of gametocytes in the bone marrow and spleen appears to be a common feature of many *Plasmodium* species (*18*–*20*), and gametocytes can develop in erythroid precursors (*21*, *22*). Asexual erythrocytic stages of some *Plasmodium* species also develop in the extravascular erythropoietic niches (*2*, *23*). Asexual replication in the erythropoietic niches can protect parasites from drug treatment and provide a reservoir for recrudescence, while the extravascular sexual development is essential for the continuation of the malaria life cycle (*21*). Yet, it remains unclear whether those phenotypes are mainly driven by the younger host cells encountered in those organs or by organ-specific factors. Uncovering the mechanistic basis for those phenotypes has been hampered by the complex cellular composition of erythropoietic niches, which is difficult to disentangle in bulk approaches such as RNA sequencing (RNA-seq). In contrast, advances in single-cell profiling have enabled the analysis of thousands of individual nonsynchronous cells in parallel (*24*, *25*).

Here, we set out to decipher the mechanisms underlying the unique parasite phenotypes in erythropoietic niches, focusing on the influence of host organ and host cell on parasite behavior. To this end, we performed comprehensive, quantitative flow cytometry and dual single-cell RNA sequencing (scRNA-seq) analysis of *P. berghei* in the spleen, bone marrow, and blood of infected mice to profile the cell and transcriptional signatures of host-parasite interactions across these niches. With this approach, we uncover transcriptional adaptation of *P. berghei* to its environment and demonstrate that the host cell rather than host organ shapes parasite behavior.

## RESULTS

### Quantitative analysis of *P. berghei* infection in the blood and erythropoietic tissues

To obtain an initial quantitative understanding of parasite distribution across host cells and organs, we followed the course of a *P. berghei* blood stage infection over 3 days by flow cytometry. We gated on RBCs as CD45^−^/Ter119^+^ cells in the spleen, bone marrow, and blood, and, within this subpopulation, we further defined three cell populations: extravascular reticulocytes expressing both CD71 and CD44 (CD44^+^/CD71^+^), intravascular reticulocytes expressing only CD71 (CD71^+^), and mature normocytes that lack both of these markers (Fig. 1A and fig. S1, A and B) (*7*, *21*). The RBC distribution differed notably between organs, with the highest proportion of reticulocytes in the bone marrow, followed by spleen, whereas only about 3.5% of all RBCs in the blood were reticulocytes (fig. S1B). At all three time points, parasitemia was highest in the spleen and lowest in the bone marrow (fig. S1C). In particular, splenic and blood intravascular reticulocytes were highly parasitized (Fig. 1B). We detected infected and uninfected CD44^+^ RBCs in the blood; however, their overall proportion in the blood was very low (less than 0.02%), further supporting CD44 as a marker for predominately extravascular RBCs (Fig. 1C and fig. S1, A and B).

To assess whether parasites were enriched in specific RBC subsets, we quantified the fraction of each host cell type in infected red blood cells (iRBCs) for each organ (Fig. 1C). We then generated a normalized fraction for each cell type and organ to account for the different frequencies of host RBC types across organs. This normalized fraction was calculated as the log fold change of host cell fraction in iRBCs over host cell fraction in all RBCs (Fig. 1C). Values greater than 0 indicate that a host cell is enriched in iRBCs compared to its fraction in total RBCs, likely caused by preferential parasite invasion of this cell type. In contrast, values smaller than 0 indicate that this cell type is less infected than would be expected given its fraction in total RBCs. In the spleen and blood, this normalization revealed a relative enrichment of intravascular reticulocytes in iRBCs, in line with the previously described preference of *P. berghei* for reticulocytes (Fig. 1C) (*16*, *17*). Although the spleen and bone marrow have high numbers of CD44^+^ extravascular reticulocytes, only a small fraction of these was infected in initial days of the time course. This suggests that at the onset of infection, *P. berghei* does not have access to the extravascular erythropoietic niches. However, after day 3 (spleen) or day 4 (bone marrow), the normalized fraction approached 0 (Fig. 1C), suggesting that extravascular reticulocytes become accessible to the parasite later during infection.

The timing of the increased infection rate of extravascular reticulocytes coincided with reported vascular leakage in those organs (*18*). To directly determine whether access to the extravascular niche correlated with vascular leakage, we analyzed sections of *P. berghei*–infected spleens by histopathology. Infected tissues exhibited histopathological signs of major splenic damage, including increasing levels of hemorrhage, necrosis, inflammation, as well as white pulp hyperplasia and lesions at later stages of infection (Fig. 1D and table S1). Immunofluorescence staining of spleen sections additionally confirmed that parasites were initially restricted to the vasculature, and extravascular parasites were only detected 3 to 4 days postinfection (dpi) as the vasculature becomes more disrupted and fragmented (Fig. 1E and fig. S2).

The *P. berghei* line used in this experiment expresses the fluorescent reporter mCherry under the control of the *hsp70* promoter, which has previously been reported to increase in expression as the parasite matures (*26*). We thus used mCherry expression levels as a proxy for parasite maturation and found that young parasite stages were enriched in the spleen (Fig. 1F and fig. S1, A and D). This enrichment could either be the result of preferential RBC invasion in the spleen or of retention of young iRBCs during splenic passage as observed for *P. falciparum* ring stages (*27*). To differentiate between these two scenarios and capture parasite localization immediately after invasion, we infected mice with synchronized schizonts and quantified ring stage distribution 1 hour postinvasion (hpi). We focused on the analysis of the spleen and blood, because our initial data revealed low infection rates in the bone marrow, which likely do not contribute significantly to parasite biomass (Fig. 1B and fig. S1C). We also expanded the marker panel by addition of a DNA stain, which enabled us to differentiate enucleated, CD44^+^/DNA^−^ extravascular reticulocytes from nucleated CD44^+^/DNA^+^ erythroblasts (Fig. 1A and fig. S3A).

**Fig. 2.**
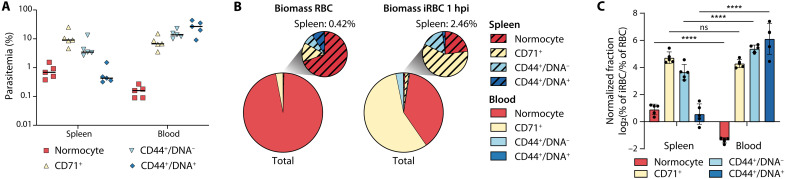
Biomass distribution of parasites right after invasion. (**A**) Parasitemia in splenic and blood RBC types 1 hour after intravenous infection with 4 × 10^7^ iRBC (*n* = 5, line at mean). (**B**) Biomass of RBC types (left) and iRBC types 1 hpi with 4 × 10^7^ iRBC (right) in total RBCs (large pie chart) and spleen RBCs (small pie chart) (*n* = 5). (**C**) Relative enrichment of host cell types in iRBC versus all RBC. Values > 0 indicate preferential invasion of this host cell type, and values < 0 indicate decreased invasion (*n* = 5, two-way ANOVA and Sidák’s post test). Means ± SEM. ns, not significant; ****P < 0.0001.

In line with the initial time course experiment, we detected the highest parasitemia in intravascular reticulocytes. Erythroblasts in the spleen were infected even less frequently than normocytes, indicating that invasion of these early precursors is a rare event (Fig. 2A). Using cell counts, we estimated total parasite biomass and found only about 2.4% of all parasites in the spleen (Fig. 2B), much lower than what has been observed recently for *P. vivax* and *P. falciparum* during human infection (*23*). Despite the tropism of *P. berghei* for reticulocytes, a significant proportion of all parasites (37.87 ± 2.14%) was present in CD71^−^ normocytes in the blood, demonstrating that *P. berghei* is not restricted to invasion of reticulocytes (Fig. 2B and fig. S3E). Again, we detected a very low number of CD44^+^ reticulocytes in the blood (below 0.1% RBCs). Although a high proportion of these were infected, given the low absolute numbers, these contributed little to overall parasite biomass (Fig. 2B). Splenic RBCs had an approximately fivefold higher chance to be infected than circulating RBCs (2.46% splenic RBCs in iRBC versus 0.42% splenic RBCs in total RBCs). This preference can largely be attributed to the high abundance of reticulocytes in this organ, and the parasite tropism for CD71^+^ cells (calculated as normalized fraction) was independent of organ (Fig. 2C). Infection rates of splenic normocytes were also higher compared to blood-derived normocytes, potentially because of the local accumulation of schizonts in the splenic sinusoids before rupture (Fig. 2C). These findings are largely independent of starting parasitemia, as similar results were obtained using a 10-fold lower inoculation dose (fig. S3, B to E). In summary, *P. berghei* preferentially invades intravascular reticulocytes in the spleen and blood, yet a substantial proportion of parasites is found within normocytes. In contrast, extravascular RBCs only become accessible to the parasite following infection-induced vascular damage.

### scRNA-seq of parasite and host cells from erythropoietic niches

**Fig. 3.**
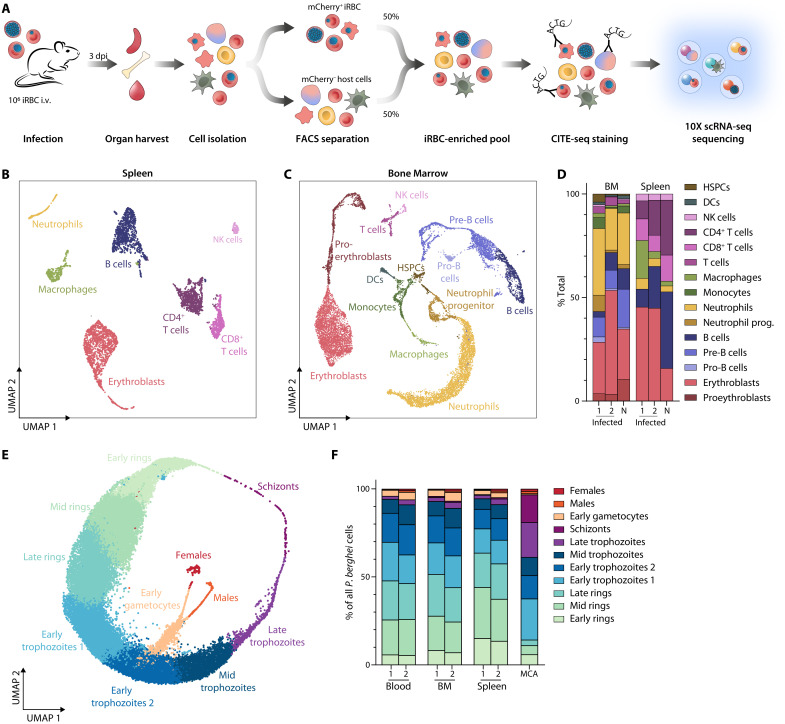
scRNA-seq analysis of *P. berghei* and host cells from the spleen, bone marrow, and blood. (**A**) Cartoon depicting experimental strategy to enrich for infected RBCs derived from the spleen, bone marrow, and blood for subsequent scRNA-seq analysis. (**B** and **C**) UMAP of the spleen (B) and bone marrow (C) host cells, colored according to clusters. (**D**) Cell type distribution in infected and naïve (N) spleen and bone marrow host cells (infected, *n* = 2; naïve, *n* = 1). (**E**) UMAP of *P. berghei* cells. (**F**) Relative proportion of parasite clusters across organs. MCA, Malaria Cell Atlas; BM, bone marrow (*n* = 2 per organ; MCA, *n* = 1).

We hypothesized that *Plasmodium* parasites would respond to the different environmental conditions encountered in these diverse host organs and cells and thus investigated transcriptional changes of parasite and host cells in the different compartments by scRNA-seq. We enriched for *P. berghei*–infected splenic, bone marrow, and blood cells by flow sorting, labeled surface CD71 and CD44 expression with barcoded antibodies [cellular indexing of transcriptomes and epitopes by sequencing (CITE-seq) (*28*)], and analyzed host and parasite transcriptomes by droplet-based scRNA-seq (Fig. 3A and fig. S4) (*29*). After removal of low-quality cells, the dataset consisted of 14,953 spleen, 13,509 bone marrow, and 12,973 blood cells from two mice, which included a total of 13,128, 7132, and 12,960 *P. berghei*–infected cells, respectively (table S2). Additional 2746 spleen, 8500 bone marrow, and 66 blood cells were obtained from one uninfected control mouse (table S2). For all samples, the mean number of RNA transcripts [unique molecular identifier (UMI)] and the mean number of genes detected per cell were comparable to or higher than in the previously published Malaria Cell Atlas (MCA), indicating high sample quality (table S2).

The cell pool for scRNA-seq included a mixture of host cells from the bone marrow and spleen. To identify erythropoietic precursors in these cells, we performed an initial analysis of these host cells by graph-based clustering of splenic and bone marrow host cell transcriptomes, followed by annotation of these clusters based on marker gene expression and public reference datasets (*24*, *30*). This approach identified 7 (spleen) and 13 (bone marrow) distinct cell types that were visualized by uniform manifold approximation and projection (UMAP) (Fig. 3, B and C, fig. S5, and table S3). Splenic clusters included CD4^+^ and CD8^+^ T cells, mature B cells, macrophages, neutrophils, erythroblasts, and natural killer (NK) cells. Bone marrow clusters consisted of immature and mature B cells, hematopoietic stem and progenitor cells (HSPCs), T cells, NK cells, proerythroblasts and erythroblasts, dendritic cells, monocytes, macrophages, neutrophil precursors, and neutrophils (Fig. 3, B and C, and fig. S5). We found a (nonsignificant) trend for an expansion of erythroblasts in infected spleens, which might indicate stress erythropoiesis (Fig. 3D). For the analysis of parasites within erythroid cells, we then selected those cells from the dataset that either fell into the erythroblast cluster or were designated RBCs based on their low number of detected host genes.

We integrated the *P. berghei* datasets from the spleen, bone marrow, and blood with the MCA (*25*). Graph-based clustering of this combined dataset yielded 12 clusters, 11 of which could be annotated on the basis of correlation with the MCA and identification of conserved marker genes (fig. S6, A to C). One cluster (cluster 7) with ambiguous marker genes and very low transcripts per cell was excluded as putative artifact (fig. S6, A to D, and table S4). Using UMAP visualization, most parasite cells arranged in circular fashion following the asexual cycle, whereas one cluster corresponding to early gametocytes diverged from early trophozoites and led to female and male gametocyte clusters (Fig. 3E). The dataset was enriched toward ring and early trophozoite stages because of the early time point of the harvest and the semisynchronicity of *P. berghei* infections (Fig. 3, E and F). Confirming the flow data, the spleen harbored more ring stages than bone marrow and blood (Fig. 3F and fig. S6E). Comparing transcription profiles across organs, the only notable differential gene expression was detected in late trophozoites, where late-stage markers such as *msp1*, *sera1*, *ron4*, and *msrp2* were down-regulated in the blood (fig. S6F and table S5). These differences probably reflect the sequestration of more mature trophozoites and schizonts in the spleen and bone marrow compared to the blood (*31*).

### Identification of CD71 as a host cell receptor for *P. berghei* invasion into reticulocytes

**Fig. 4.**
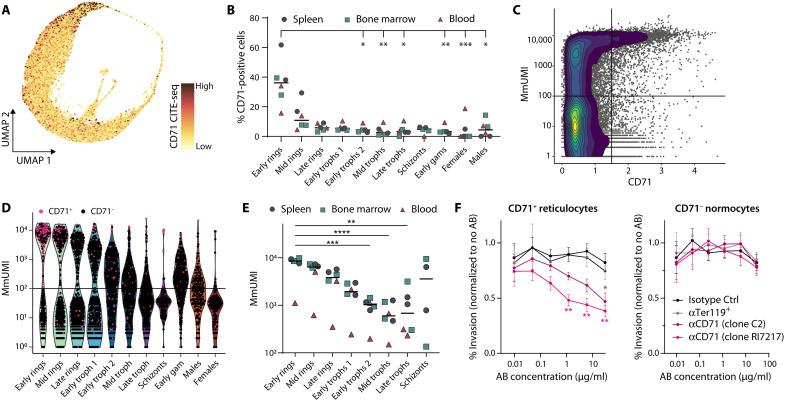
*P. berghei* has a bimodal invasion pattern into either CD71-positive reticulocytes or normocytes. (**A**) UMAP of *P. berghei* cells colored by CD71 surface protein levels as measured by CITE-seq. (**B**) Proportion of CD71-positive cells per cluster in each sample. Organs are indicated by color [*n* = 6 (2 per organ), line at mean, Friedman test and Dunn’s post test]. (**C**) Host RNA levels, quantified as mouse-derived UMI (MmUMI), plotted against CD71 surface protein levels as measured by CITE-seq. Horizontal and vertical lines indicate thresholds separating CD71-positive (CD71 > 1.5) from CD71-negative cells and reticulocytes (MmUMI >100) from normocytes, respectively. (**D**) Host RNA levels across stages. Cells are randomly subsampled to 1000 cells per stage. Color of dots indicates CD71-positive (pink) and CD71-negative cells (black). (**E**) Average host RNA levels per cluster in each sample. Organs are indicated by color [*n* = 6 (2 per organ), line at mean, Friedman test and Dunn’s post test]. (**F**) Invasion of merozoites in CD71-positive reticulocytes (left) or CD71-normocytes (right) in the presence of anti-CD71 antibody (two different clones), anti-Ter119 antibody, or isotype control. Invasion was normalized to no antibody (AB) treatment and significance tested against isotype control (*n* = 6, means ± SEM, two-way ANOVA and Dunnett’s post test). (B, C, and E) *P < 0.05; **P < 0.01; ***P < 0.001; ****P < 0.0001.

We next aimed to classify the infected host cells to define host cell–dependent differences in parasite transcriptomes. We first quantified the surface expression of CD71 and CD44 based on the number of reads detected from the barcoded antibodies used in CITE-seq. While we did not detect any reads matching to the antibody barcode targeting CD44, probably due to the low expression of this marker, we could detect CD71 surface expression (as defined by a normalized CD71 signal > 1.5) on roughly 11% of all cells (Fig. 4A). CD71 surface expression was almost exclusive to reticulocytes infected with early rings and was rapidly lost in further developmental stages, suggesting reticulocyte comaturation with parasite development (Fig. 4, A and B). Previous studies have shown that reticulocyte maturation to normocytes is also accompanied by a loss of RNA content (*6*). Using the number of detected host transcripts [*Mus musculus* UMI (MmUMI)] as a proxy for host RNA content per cell, we found that CD71^+^ reticulocytes contained a high amount of RNA (Fig. 4C). In line with previous observations, loss of CD71 surface expression preceded host RNA degradation (Fig. 4C) (*6*). We thus used MmUMI as a more sensitive marker to determine RBC maturation status and defined host cells with 100 MmUMI or more as reticulocytes and cells with less than 100 MmUMI as normocytes (Fig. 4C).

Plotting host RNA content against parasite maturation status revealed that early rings were almost exclusively detected in either reticulocytes with a high RNA content (>1000 MmUMI) or in normocytes (<100 MmUMI) (Fig. 4D). The lack of early rings in reticulocytes with intermediate RNA content (100 < MmUMI < 1000) suggested a bimodal invasion pattern based on availability of specific host cell receptors. The average host RNA content in infected reticulocytes was comparable among organs but decreased with parasite development, indicating a comaturation of parasite and host cell (Fig. 4E).

Most reticulocytes harboring early rings were also CD71^+^ (Fig. 4D), and recent work has revealed that CD71 is an entry receptor for the human malaria parasite *P. vivax* (*13*). We thus hypothesized that CD71 might also serve as receptor for *P. berghei* reticulocyte invasion. To test this hypothesis, we pretreated naïve mouse blood with anti-CD71 antibodies before ex vivo invasion with *P. berghei* merozoites and measured invasion efficiency into normocytes and reticulocytes by flow cytometry. Two different monoclonal anti-CD71 antibodies blocked *P. berghei* invasion into reticulocytes in a dose-dependent manner but did not inhibit invasion of normocytes (Fig. 4F and fig. S7). Furthermore, the invasion rate into reticulocytes correlated negatively with the intensity of the blocking antibody staining [Pearson, *r* = −0.6033 (clone C2) and *r* = −0.6741 (clone RI71217), *n* = 36, *P* ≤ 0.0001] (fig. S7C). These results strongly suggest that *P. berghei* requires CD71 as receptor for reticulocyte invasion yet uses an alternative receptor for normocyte invasion.

### *P. berghei* transcriptionally adapts to host cell maturation

**Fig. 5.**
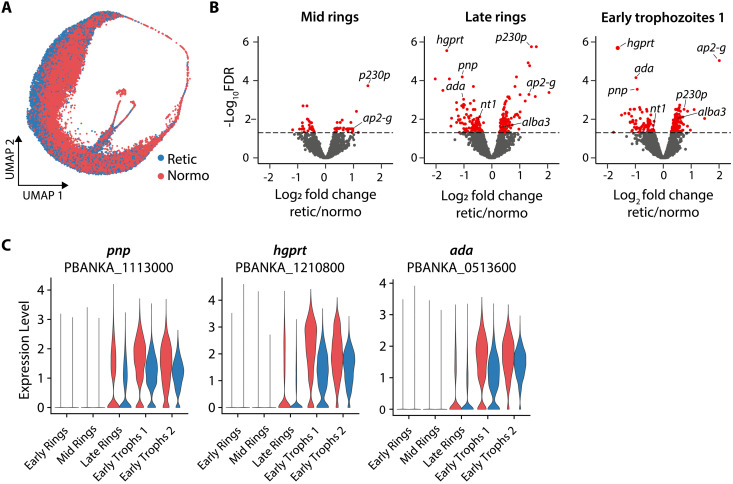
*P. berghei* adapts transcriptionally to its host cell maturation status. (**A**) UMAP of *P. berghei* cells colored by host cell (red, normocytes; blue, reticulocytes). (**B**) Volcano plot of differential gene expression in reticulocyte-infecting parasites versus normocyte-infecting parasites in selected stages. Red, genes passing the false discovery rate (FDR) threshold. (**C**) Expression level of genes of the purine salvage pathway in early parasite stages, separated by host cell (red, normocytes; blue, reticulocytes). Retic, reticulocytes; Normo, normocytes.

Parasite cells separated on the UMAP according to their host cell, indicating transcriptional adaptation to the different host environments (Fig. 5A). We analyzed differential gene expression in early rings to early trophozoites, as in those stages normocyte and reticulocyte host cells could be clearly differentiated (Fig. 4D). Signatures of differential parasite gene expression according to the host cell were detected in mid ring, late ring, and early trophozoite 1 clusters (Fig. 5B and table S6). Specifically, purine nucleoside phosphorylase (*pnp* PBANKA_1113000), hypoxanthine-guanine phosphoribosyltransferase (*hgprt* PBANKA_1210800), adenosine deaminase (*ada* PBANKA_0513600), and the nucleotide transporter 1 (nt1 PBANKA_1360100) were significantly up-regulated in normocyte-infecting parasites (Fig. 5, B and C). These genes are all part of the purine salvage pathway to synthesize purine nucleotides from host metabolites, and their up-regulation might compensate for the limited abundance of those metabolites in normocytes compared to reticulocytes (*15*).

**Fig. 6.**
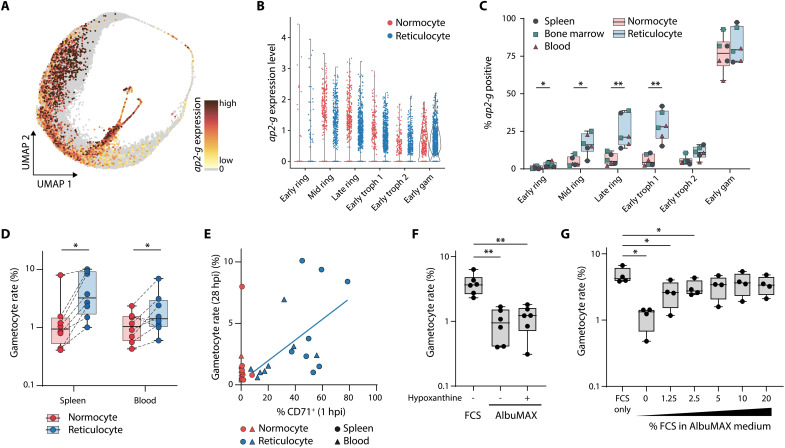
*P. berghei* sexual development occurs preferentially in reticulocytes. (**A**) UMAP of *P. berghei* colored by AP2-G expression strength. (**B**) *ap-2g* expression level in early stages, separated by host cell. (**C**) Proportion of *ap-2g*–positive cells in early stages, separated by host cell (box plot color). Individual dots are colored by organ (*n* = 6). (**D**) Gametocyte conversion rate after 28 hours of ex vivo culture of normocyte- or reticulocyte-enriched iRBC isolated from the spleen or blood. Samples from one organ were matched across host cell (*n* = 8, two-way ANOVA and Tukey’s post test). (**E**) Correlation between proportion of CD71^+^ cells 1 hpi and subsequent gametocyte rate after 28 hours of ex vivo culture. Same samples as in (D). (**F**) Gametocyte rate after ex vivo culture in nutrient-rich or nutrient-poor medium (*n* = 6, one-way ANOVA and Dunnett’s post test). (**G**) Gametocyte rate after ex vivo culture in minimal medium supplemented with varying concentrations of FCS (*n* = 4, one-way ANOVA and Dunnett’s post test). (C, D, F, and G) Center line, median; box limits, upper and lower quartiles; whiskers, minimum to maximum range; all points shown. *P < 0.05; **P < 0.01.

One of the most notable genes up-regulated in reticulocyte-infecting parasites was *ap2-g*, a conserved transcription factor activating the first set of sexual stage markers in *Plasmodium* (Fig. 5B) (*32*, *33*). Other gametocyte-related genes such as *alba3* and *p230p* (*34*) also exhibited higher expression in reticulocyte-infecting parasites, supporting a link between sexual commitment and reticulocyte invasion (Fig. 5B). On the UMAP visualization, *ap2-g*–positive cells were most prevalent in early ring to early gametocyte stages and appeared to follow reticulocyte distribution (Figs. 6A and 5A). Overall *ap2-g* expression levels per parasite stage did not differ between normocyte- and reticulocyte-infecting parasites (Fig. 6B). However, the proportion of early parasite stages positive for AP2-G was significantly higher in reticulocytes compared to normocytes, independent of host organ (Fig. 6C). This data suggest that for *P. berghei*, the rate of sexual commitment is dependent on the host cell, not the organ.

### A serum factor triggers sexual differentiation upon reticulocyte invasion in *P. berghei*

To independently investigate this observation, we quantified gametocyte rates in different host cells ex vivo. Using a gametocyte reporter line expressing green fluorescent protein (GFP) constitutively and red fluorescent protein (RFP) in gametocytes [*Pb*GFP_CON_/RFP_GAM_ (*35*)] (*21*), we magnetically sorted freshly invaded RBCs into a CD71-enriched reticulocyte and a CD71-depleted normocyte fraction and quantified the proportion of parasites that developed into gametocytes (gametocyte rate) after 28 hours of ex vivo culture by flow cytometry (fig. S8, A to E). As expected, parasitemia was higher in reticulocytes compared to normocytes. The rate of sexual commitment was also significantly increased in reticulocytes compared to normocytes from the same sample (Fig. 6D and fig. S8F). Gametocyte rates in the reticulocyte fraction correlated with the proportion of CD71^+^ cells after purification (Pearson, *r* = 0.53, *n* = 16, *P* = 0.0332), although the latter might be underestimated due to steric hindrance of CD71 binding by the anti-CD71 antibodies used for magnetic cell purification (Fig. 6E). Notably, there was no significant difference in the gametocyte rate between the spleen and blood (Fig. 6D). In summary, both scRNA-seq and ex vivo data indicate that sexual commitment is higher in reticulocytes than in normocytes, independent of host organ.

We hypothesized that purine levels serve as metabolic triggers for sexual commitment, because down-regulation of the purine salvage pathway has been previously correlated with a decrease in gametocytemia, and we found purine salvage enzymes to be up-regulated in normocytes (*36*). To test gametocyte conversion in different metabolic environments, we thus cultured young ring stages in either nutrient-rich standard medium supplemented with fetal calf serum (FCS) or in minimal medium consisting of RPMI-AlbuMAX for 28 hours and measured gametocytemia by flow cytometry (fig. S9, A and B). In minimal medium, we observed a basal gametocyte rate of about 1%, which could not be increased by the addition of the purine precursor hypoxanthine (Fig. 6F). In contrast, in nutrient-rich medium containing FCS, we saw a notable increase of sexual commitment within the same cycle (Fig. 6F and fig. S9, A and B). This difference in gametocyte rates was not due to delayed gametocyte maturation, as the median RFP fluorescence of gametocytes did not differ between conditions (fig. S9C). The basal gametocyte rate increased with increasing FCS or mouse serum content in the medium in a dose-dependent manner up to 3 to 4%, indicating that a specific molecule (or a combination) present in serum triggers sexual commitment of early rings in *P. berghei* within the same cycle (Fig. 6G and fig. S9D). Notably, the gametocyte rate of normocyte-invading parasites that were cultured in rich medium remained at the basal level of 1% (Fig. 6D), suggesting that this external trigger is only active on reticulocyte-invading parasites. In summary, our data demonstrate that for *P. berghei*, a substantial proportion of gametocytes are formed as a result of nutrient-dependent same cycle sexual commitment (SCC) that is triggered upon invasion of metabolically active reticulocytes.

## DISCUSSION

A series of recent studies have established erythropoietic niches in the extravascular parenchyma of bone marrow and spleen as major sites of *Plasmodium* asexual and sexual development. Specific parasite adaptations to those niches include cryptic parasite replication cycles and increased gametocyte formation (*2*, *18*–*21*, *23*). Understanding the interactions between *Plasmodium* and the erythropoietic system should improve our ability to interfere with this extravascular parasite reservoir. Here, we present a detailed single-cell analysis of host parasite interactions in erythropoietic niches using the rodent malaria model *P. berghei*.

*P. berghei* has a known tropism for reticulocytes (*16*, *17*); consistent with this tropism, we observed a significant enrichment of CD71^+^ reticulocytes in iRBCs. This host cell tropism was independent of organ, and the observed enrichment of parasites in the spleen could be attributed to the higher abundance of CD71^+^ reticulocytes in this organ. However, despite the clear preference for splenic reticulocytes, we found most of the parasite biomass in circulation and over a third of all parasites invaded circulating normocytes. These findings are in contrast with a recent study that compared parasite biomass of *P. vivax* between spleen and blood and estimated that more than 93% of all parasites were present in the spleen (*23*). In further contrast to previous findings in *P. falciparum* and *P. vivax* infections (*19*), we did not observe major asexual replication of *P. berghei* parasites in the bone marrow. Our study thus highlights critical differences between *Plasmodium* species that should be taken into account when investigating blood stage biology and, in particular, the tropism of parasites in different host organs in the rodent model.

Using a combination of scRNA-seq, CITE-seq, and host RNA content, we were able to define parasite transcriptomes in different host cells, including the youngest ring stages. This analysis suggested that *P. berghei* exhibits a bimodal invasion pattern into either early CD71^+^ reticulocytes or fully matured normocytes. Invasion inhibition assays revealed that the transferrin receptor CD71 serves as key host receptor for reticulocyte invasion by *P. berghei*. Residual invasion at high anti-CD71 antibody levels may be due to rapid CD71 recycling, resulting in a release of CD71 receptor onto the surface of the reticulocyte, after staining with blocking antibody and before invasion, that could serve as entry receptor for merozoites (*37*, *38*). Alternatively (or additionally), incomplete steric hindrance of the selected monoclonal antibodies for parasite ligand binding or the presence of alternative entry receptors might contribute to residual invasion activity. The parasite ligand binding to CD71 remains to be determined. Notably, the protein *Pb*maLS_05 (*PBANKA_140100*) has been reported to be required for maintaining *P. berghei* reticulocyte preference (*39*). Additional candidates are members of the superfamily of reticulocyte binding–like (RBL) proteins. In rodent malaria parasites, the RBL family has expanded to include multiple members (known as Pb235 proteins in *P. berghei*), which are implicated in host cell tropism (*40*, *41*). One of the two *P. vivax* RBL proteins, PvRBP2b, has recently been identified as the CD71 ligand required for reticulocyte invasion (*13*). It is currently also unclear by which receptor-ligand interaction(s) *P. berghei* invades normocytes. RBCs undergo extensive surface remodeling during maturation yet lack capability for de novo gene expression (*4*, *5*). The invasion of normocytes could thus be due to surface protein loss, steric changes, and increasing accessibility of the receptor or to conformational changes of the receptor during erythrocyte maturation. Alternatively, changes in biophysical properties of the RBC such as deformability might enable productive invasion (*6*). *P. falciparum* also invades RBCs via a broad spectrum of invasion receptors, and invasion efficiency is modulated by membrane mechanics (*42*).

Last, we identified several parasite phenotypes that are determined by host cell, not organ tropism. *P. berghei* asexual parasites in normocytes show increased expression of the genes for the purine salvage enzymes ADA, HGPRT, and PNP compared to parasites in reticulocytes. This up-regulation of metabolic activity likely compensates for the reduced levels of purine precursors in normocytes; i.e., up-regulation of purine-salvage genes may represent a response to the metabolically scarce normocyte environment. Transcriptional data comparing culture-adapted *Plasmodium knowlesi* to reticulocyte-restricted parasites from malaria patients suggests that such metabolic adaptation to the host cell is conserved across species (*43*) and may represent a novel avenue for host targeted antimalarial interventions.

Among the most significantly up-regulated genes in reticulocyte-invading parasites was the transcription factor *ap2-g*, an essential component of the sexual commitment pathway in *Plasmodium* parasites (*32*, *33*). Ex vivo assays using gametocyte reporter parasites demonstrated increased gametocyte levels in reticulocytes compared to normocytes. Previous studies using *P. berghei* and *P. chabaudi* (another rodent *Plasmodium* species) found an increased gametocyte rate under conditions of stress erythropoiesis induced by pretreatment of mice with the hemolytic hydrazine derivative phenylhydrazine (*35*, *44*–*46*). Yet, it remained unclear whether this increased sexual conversion is caused by the hyperparasitemia observed in phenylhydrazine-treated mice, host factors released during stress erythropoiesis, or whether it is specifically triggered by invasion of reticulocytes. Our study presents direct evidence that a substantial proportion of sexual commitment in *P. berghei* is the result of reticulocyte invasion during natural infection.

Exposure of parasites to various nutritional conditions suggested that sexual commitment in reticulocytes, but not in normocytes, is triggered by a serum factor. This unknown serum factor may act as an environmental sensor and activator of sexual commitment, after it is imported in and/or metabolized by reticulocytes only. A recent study suggests that limiting parasite *S*-adenosylmethionine (SAM) and *S*-adenosylhomocysteine (SAH) levels results in *ap2-g* activation and sexual commitment both in *P. falciparum* and *P. berghei* (*47*). It is conceivable that the factor triggering sexual commitment in nutrient-rich environments is associated with SAM/SAH metabolism. Yet, further experimental work is required to identify the serum factor and test this hypothesis.

As our ex vivo assays were done with parasites after invasion, the data also demonstrate that activation of sexual commitment through this serum factor is triggered in the same cycle as the formation of gametocytes occurs. Such SCC has recently been demonstrated both in *P. berghei* and *P. falciparum* in an artificial system relying on experimentally induced *ap2-g* expression (*48*, *49*). However, next cycle commitment (NCC) is the predominant mode of sexual commitment in *P. falciparum*. This pathway has been well characterized and can be activated in trophozoites via nutrient depletion and activation of *ap2-g* via the nuclear factor gametocyte development 1 (GDV1) (*50*, *51*). Recent studies have identified the serum phospholipid lysophosphatidylcholine as an environmental sensor and repressor of NCC in the human malaria parasite, *P. falciparum* (*51*). The NCC pathway via nutrient depletion and GDV1 is not functional in *P. berghei* (*51*). Instead, our data demonstrate that SCC triggered by addition of a serum factor can account for most of the sexual commitment in *P. berghei.* Whether this mode of sexual commitment is also operational in *P. falciparum* beyond genetically inducible activation remains unclear.

In this study, we unravel complex host-parasite interactions in vivo on a single-cell level to provide fundamental biological insights and identify new phenotypes of *P. berghei* that are dependent on the maturation status of the host cell. Our study could serve as a blueprint for similar investigations of *P. falciparum* and *P. vivax* to characterize convergent and divergent features of these human malaria parasites.

## MATERIALS AND METHODS

### Mice

All animal experiments were performed according to the guidelines defined by the Home Office and U.K. Animals (Scientific Procedures) Act 1986 and approved by the U.K. Home Office (project license P6CA91811) and the University of Glasgow’s Animal Welfare and Ethical Review Body or according to the Federation of European Laboratory Animal Science Associations (FELASA) and Gesellschaft für Versuchstierkunde/Society of Laboratory Animal Science (GV-SOLAS) guidelines and approved by the responsible German authorities (Regierungspräsidium Karlsruhe). For most experiments, female Theiler Original mice weighing 25 to 30 g were purchased from Envigo and were aged 5 to 8 weeks at the time point of infection. For investigating nutrient dependency of sexual commitment, female Swiss mice weighing 20 to 25 g were purchased from JANVIER and were aged 5 to 8 weeks at the time point of infection. For each experiment, mice were age-matched and were allocated randomly to each group.

### *P. berghei* infections

Most experiments were performed using a *P. berghei* line constitutively expressing mCherry under the control of the *hsp70* promoter (*Pb*mCherry) (*52*). To investigate gametocyte conversion rates in different host cells under varying nutrient conditions, we used the previously published *P. berghei* ANKA *Pb*GFP_CON_/RFP_GAM_ line that expresses green fluorescent protein (GFP) under the control of the constitutive ef1a promoter and RFP under the gametocyte-specific PBANKA_101270 promoter (*21*). Infections were initiated by intraperitoneal infection of donor mice with ~200 μl of thawed cryostocks. Depending on the experiment, recipient mice were inoculated intravenously with mixed blood stages obtained from donor mice or with synchronized schizont stages obtained as detailed below. Parasitemia was monitored by Giemsa-stained blood smears or flow cytometry. At indicated time points after infection, mice were bled by cardiac puncture under terminal anesthesia and culled by cervical dislocation.

### Blood and tissue collection

Blood was collected from mice under terminal anesthesia by cardiac puncture using a heparinized syringe. For organ collection, mice were subsequently culled by cervical dislocation, spleen, femur, and tibia harvested, and the organs were placed into 1 ml of fluorescence-activated cell sorting (FACS) buffer [0.2 to 0.5% bovine serum albumin (BSA)/phosphate-buffered saline (PBS), sterile-filtered] on ice. Bone marrow was released by removing the ends of the bones with sharp scissors and perfusing the bones with 1 ml of cold FACS buffer. Spleen and bone marrow were dissociated by gently straining the tissues through a 40-μm cell strainer using the plunger of 5-ml syringe into 50-ml tubes. Blood (50 to 100 μl) was filtered through a 40-μm cell strainer into 50-ml tubes. Cell strainers were flushed with 25 ml of FACS buffer, and cells were pelleted by centrifugation for 10 min at 4°C. Blood, spleen, and bone marrow cells were resuspended in 20, 10, and 1 ml of cold FACS buffer, respectively. Cells were counted in a Neubauer counting chamber.

### Organ preparation for histology and immunofluorescence

Spleens for histological analysis were cut in half transversely and placed in 4% paraformaldehyde (PFA) for 5 hours, before transfer to 15% (w/v) sucrose in PBS for 2 hours and then 30% (w/v) sucrose overnight (all at 4°C) for fixation and cryoprotection. Spleens were embedded in OCT (CellPath) using dry ice and isopentane and stored at −80°C. Sections were cut at 3 to 5 μm on a cryotome (Thermo Fisher Scientific) using C35 carbon steel blades (Feather) on Superfrost Plus slides (Thermo Fisher Scientific). Cut sections were wrapped in tin foil and stored at −80°C until staining. For hematoxylin and eosin staining, sections were removed from the freezer and left wrapped in foil for 20 min, unwrapped, and then left for a further 10 min at room temperature (RT). Sections were fixed in ice-cold acetone/ethanol (75%/25%) for 10 min, air-dried for 10 min, and then placed in distilled water for 3 min before placement in acidified Harris hematoxylin (CellPath) for 3 min. Excess stain was removed by brief introduction to a running tap water bath, before two dips in 1% acetic acid in ethanol solution to differentiate, further rinsing in tap water, 30 s in Scott’s Tap Water substitute (Atom Scientific), one final tap water rinse, 10 dips in 70% ethanol, and then 2 min of incubation in 1% alcoholic eosin Y stain (CellPath). Slides were dehydrated gradually by incubation in 90% ethanol twice, 100% ethanol twice, and last, xylene twice before mounting with a coverslip and DPX (CellPath) mountant. Slides were assessed for features from slide scans taken with a Leica Aperio Versa. Images (100×) were taken on an Olympus BX43F light microscope with DP22 camera (Olympus). For immunofluorescence assays, sections were removed from the freezer and left wrapped in foil for 20 min at RT, before rehydration in 1× PBS for 3 min. Sections were drawn around with a hydrophobic pen; 4% PFA was added to each section and incubated in a humid chamber for 3 min, before washing in PBS twice for 3 min. Sections were permeabilized with 0.2% Triton X-100 for 10 min and washed again in PBS twice for 3 min. Sections were blocked with 2.5% normal goat serum (Vector Laboratories) supplemented with 2.5% normal mouse serum (Invitrogen) for 1 hour at RT. Sections were then incubated with rabbit anti-CD31 (1.25 μg/ml) (NB100-2284, Novus Biologicals) overnight at 4°C. The day after, sections were rested at RT for 30 min before washing thrice with PBS for 3 min each and incubating with 1:200 goat anti-rabbit immunoglobulin G (IgG)–Alexa Fluor 647 (A21245, Invitrogen) for 1 hour. Last, sections were washed in PBS twice, stained with 2.5 μM 4′,6-diamidino-2-phenylindole (Sigma-Aldrich), washed with PBS, then treated with TrueView autofluorescence quencher (Vector Laboratories), washed with PBS, and mounted and coverslipped with Vectashield Vibrance (Vector Laboratories). Images were taken on a Nikon A1R Ti2 confocal microscope at 60× with immersion oil (Nikon).

### Flow cytometry analysis

Approximately 1 × 10^6^ to 1 × 10^7^ cells were transferred to a 96-well plate, pelleted for 2 min at 400*g*, resuspended in 100 μl of FACS buffer and 5 μl of TruStain FcX (anti-mouse CD16/32) (BioLegend, catalog no. 101320), and incubated for 10 min on ice. Antibodies and DNA stains were added, and cells were stained for 30 min on ice. Cells were pelleted (2 min at 400*g*) and washed twice with 200 μl of FACS buffer. After resuspension in 200 μl of FACS buffer, cells were analyzed on a MACSQuant VYB flow cytometer or a BD FACSCelesta. Data were analyzed using FlowJo software (v. 10.7.2).

### Ex vivo maturation of schizonts and parasite stage separation via density gradient

To obtain mature schizonts, donor mice with 1 to 2% parasitemia were bled, and the blood was cultured for 16 to 20 hours in 25 to 30 ml of 20% FCS/RPMI at 5% O_2_/5% CO_2_. Schizonts were purified by density gradient centrifugation as described previously (*53*). Briefly, the culture was layered on 5 ml of 55% Nycodenz/PBS, centrifuged for 20 min at 600*g* with reduced acceleration and deceleration, and schizonts were collected from the interphase. For depletion of late stages from cell suspensions, cells were separated using the same density gradient but retaining the pellet containing ring stages and uninfected RBCSs. Either fraction was collected, washed with 10 ml of 20% FCS/RPMI, counted, and further processed as described in the individual sections.

### Parasite distribution across host organs and host cells

For the time course, mice were intravenously inoculated with 1 × 10^6^ mixed-stage iRBC. Blood and organs were harvested 2, 3, and 4 dpi as described above. Cells were processed for flow cytometry and stained with BV421 anti-mouse CD45 (BioLegend, catalog no. 103134), fluorescein isothiocyanate (FITC) anti-mouse CD71 (BioLegend, catalog no. 113806), phycoerythrin (PE)/Cy5 anti-mouse/human CD44 (BioLegend, catalog no. 103010), PE/Cy7 anti-mouse Ter119 (BioLegend, catalog no. 116222) at a 1:100 dilution, and eBioscience Fixable Viability Dye eFluor 506 (Invitrogen, catalog no. 65-0866-14) at a 1:1000 dilution.

For assessing parasite invasion, mice were intravenously inoculated with 4 × 10^6^ or 4 × 10^7^ purified schizonts. Blood and organs were harvested 1 hour after infection and processed for flow cytometry as described above. Cells were stained with Hoechst (Abcam, ab228551) at a 1:5000 dilution and BV510 anti-mouse CD45 (BioLegend, catalog no. 103137), FITC anti-mouse CD71 (BioLegend, catalog no. 113806), PE/Cy5 anti-mouse/human CD44 (BioLegend, catalog no. 103010), and PE/Cy7 anti-mouse Ter119 (BioLegend, catalog no. 116222) at a 1:100 dilution.

### CD71 invasion assay

To obtain host cells for in vitro invasion, 30 to 50 μl of blood was harvested from a naïve mouse, resuspended in 10 ml of 0.5% BSA/PBS, and seeded in a 96-well plate at 5 × 10^6^ cells per well. Unspecific bindings were blocked by addition of 5 μl of TruStain FcX (anti-mouse CD16/32) (BioLegend, catalog no.101320) and incubation for 10 min on ice. Cells were stained in 50 μl of total volume for at least 30 min on ice with PE/Cy7 anti-mouse Ter119 (BioLegend, catalog no. 116222) (1:50 dilution) and BV510 anti-mouse CD71 (clone RI7217; BioLegend, catalog no. 103145), BV510 rat anti-mouse CD71 (clone C2; BD Biosciences catalog no. 563112), or BV510 rat IgG2a (κ isotype ctrl antibody; BioLegend, catalog no. 400553) at varying concentrations (30, 6, 1.2, 0.24, 0.048, and 0.0096 μg/ml). Cells were pelleted (2 min at 400*g*) and resuspended in merosome-containing medium (see below).

In parallel, *Pb*mCherry schizonts were purified from an overnight culture by density gradient centrifugation as described above, counted, and resuspended in schizont medium. Merozoites were mechanically released by vigorous pipetting and sequential passage through a 5-μm filter (Acrodisc) and a 1.6-μm filter (Puradisc, Whatman) as described previously (*21*). Filters were flushed with schizont medium, and flow-throughs were pooled and diluted to 1 ×10^7^ ruptured schizonts/ml. A total of 200 μl (2 ×10^6^ cells) per well was used to resuspend CD71-blocked host cells. After centrifugation for 10 min at 1200g, cells were incubated for 3 hours at 37°C, 5% O_2_/5% CO_2_. Cells were processed for flow cytometry as described above, staining with FITC anti-mouse CD71 (BioLegend, catalog no. 113806) at a 1:100 dilution and Hoechst (Abcam, ab228551) at a 1:25,000 dilution. We noted that prestaining reticulocytes with increasing concentrations of BV510-coupled blocking anti-CD71 antibody before invasion resulted in a titratable decrease of FITC-CD71 staining after invasion, indicating that the binding of the FITC-coupled detection antibody was inhibited (fig. S7, B and C). To identify CD71^+^ cells, we therefore chose a gate to include all cells that were either BV510^+^, FITC^+^, or BV510^+^/FITC^+^ (fig. S7, A and B).

### Separation and ex vivo maturation of infected reticulocytes and normocytes

Donor mice infected with >1% *Pb*GFP_CON_/RFP_GAM_ parasites were bled, and late stages including gametocytes were depleted by density gradient purification as described above. The pellet containing ring stages was washed once in 10 ml of schizont medium (5 min, 500*g*), resuspended in 30 ml of schizont medium, and cultured for approximately 20 hours on a shaker at 37°C, 5% O_2_/5% CO_2_. Mature schizonts were purified by density gradient purification, and 5 × 10^6^ schizonts were intravenously injected into recipient mice. One hour after infection, mice were culled, and blood and spleen were collected as described above. Cell pellets were resuspended in 10 ml of 0.5% BSA/PBS, leukocyte-depleted by filtering through prewetted Plasmodipur (Europroxima) filters, and the remaining late stages and gametocytes were removed by density gradient centrifugation as described above. The pellet containing freshly invaded ring stages was washed once in 10 ml of magnetic-activated cell sorting (MACS) buffer (0.5% BSA/0.2 mM EDTA/PBS) and resuspended to 5 × 10^8^ cells/ml (blood) or 2.5 × 10^8^ cells (spleen). Cell suspension (400 μl) was incubated with 80 μl of TruStain FcX (anti-mouse CD16/32) (BioLegend, catalog no.101320) for 10 min on ice, followed by addition of 20 μl of purified anti-mouse CD71 antibody (BioLegend, catalog no. 113802) and further 30 min of incubation on ice. Cells were washed with 10 ml of MACS buffer (5 min at 1600 rpm) and resuspended in 800 μl of MACS buffer before addition of 200 μl of anti-rat IgG microbeads (Miltenyi Biotec, catalog no. 130-0480501). Volumes for CD71 and microbead staining were proportionally adapted in case less than 1 × 10^8^ cells were obtained from the spleen. After 15 min of incubation on ice and a second wash with 10 ml of MACS buffer, cells were resuspended in 800 μl of MACS buffer and loaded on preequilibrated LS columns (Miltenyi Biotec) placed in a magnet. The flow-through containing CD71-negative normocytes was collected, and columns were washed three times with 3 ml of MACS buffer, pooling all flow-throughs. CD71-positive reticulocytes were eluted by removing the columns from the magnet and flushing them with 5 ml of MACS buffer using the plunger. Reticulocytes and normocytes were counted, pelleted (10 min at 1600 rpm), and resuspended in schizont medium. A total of 2 × 10^7^ (normocytes) or 1 × 10^7^ cells (reticulocytes) were seeded in a 96-well plate and incubated for 28 hours at 37°C, 5% O_2_/5% CO_2_.

Aliquots of cells collected before and after MACS separation were analyzed by flow as described above after staining with BV510 anti-mouse CD71 (BioLegend, no. 103145), PE/Dazzle 594 anti-mouse CD45 (BioLegend, no. 103145), PE/Cy5 anti-mouse/human CD44 (BioLegend, no. 103010), PE-Cy7 anti-mouse Ter119 (BioLegend, no. 116222) (1:100 dilution), and Hoechst (Abcam, ab228551) (1:5000 dilution). After 28 hours of incubation, cells were analyzed by flow cytometry after staining with PE/Dazzle 594 anti-mouse CD45 (BioLegend, no. 103145), PE-Cy7 anti-mouse Ter119 (BioLegend, no. 116222) (1:100 dilution), and Hoechst (Abcam, ab228551) (1:5000 dilution). Gametocyte rates were determined by flow cytometry as percentage of RFP-positive cells among all GFP-positive parasites.

### Gametocyte conversion under varying hypoxanthine concentrations

Mature schizonts obtained from an overnight culture of *Pb*GFP_CON_/RFP_GAM_ were purified by density gradient and intravenously injected into recipient mice. Mice were bled 1 hour after invasion, and the remaining schizonts and gametocytes were removed by density gradient. The pellet was washed once in minimal medium [RPMI 1640 (Gibco, no. 52400-025) with 0.5% Albumax II (Gibco)] and resuspended to a hematocrit of 1.25% in minimal medium containing 250 μM hypoxanthine (c.c.pro) and 2 μM choline (Sigma-Aldrich). The medium was additionally supplemented with FCS (Gibco) or mouse serum obtained freshly from a naïve mouse. Parasites were cultured for 24 hours at 37°C, 5% CO_2_/5% O_2_ before analysis by flow cytometry on a BD FACSCelesta. Gametocyte rates were determined by flow cytometry as percentage of RFP-positive cells among all GFP-positive parasites.

### Cell preparation for scRNA-seq

Blood, spleen, and bone marrow were harvested from a naïve mouse or from an infected mouse 3 dpi with 1 × 10^6^ *Pb*mCherry parasites as described above. Dissociated spleen, bone marrow, and blood cells were strained a second time through a 40-μm filter and were sorted on the basis of mCherry fluorescence using a BD FACSAria IIu or BD FACSAria III, collecting both mCherry-positive and mCherry-negative cells. Per organ, 0.5 × 10^6^ cells from each mCherry-positive and mCherry-negative fraction were pooled; pelleted for 10 min at 500*g*, 4°C; and resuspended in 100 μl of 0.2% BSA/PBS. Unspecific antibody binding was blocked by adding 10 μl of TruStain FcX (anti-mouse CD16/32) (BioLegend, catalog no. 101320) to each sample and incubating for 10 min on ice. Cells (20 μl) were removed for flow analysis and remaining cells were incubated for 30 min on ice with 1 μg each of TotalSeq-A0441 anti-mouse CD71 antibody (BioLegend, catalog no. 113824) and TotalSeq-A0073 anti-mouse/human CD44 antibody (BioLegend, catalog no. 103045). Cells were washed by adding 1 ml of 0.2% BSA/PBS and pelleted for 5 min at 2800*g*, 4°C. Pellets were resuspended in 1 ml of 0.2% BSA/PBS and washed twice more. Cells were filtered through a 40-μm strainer (Flowmi) and counted on an automatic cell counter (Countess 3, Invitrogen) immediately before loading onto the 10X Chromium machine. The 10X libraries (generated following the standard 10X protocol) were sequenced on an Illumina NextSeq 550 to ~40,000 to 50,000 reads per cell with reads length of 27 for read 1 and 130 for read 2. CITE-seq libraries were prepared following a previously published protocol (*28*) and sequences on an Illumina NextSeq 550 with around 5000 reads per cell and CITE-seq adapter.

### Data analysis

#### Transcriptome mapping

The raw Illumina reads were mapped with Cell Ranger (v. 3.1.0) against a combined reference of Mus musculus (Mouse) version 93 and *P. berghei* version 3 with extended 3′ untranslated region. CITE-seq reads were processed with CITE-seq-Count (v. 1.4.3).

#### Data preprocessing and quality control

Quality control and data integration were performed in R (v. 3.6.1) using Seurat (v. 3.2.2) (*54*). As the filtered Cell Ranger count matrices did not include early ring stages within normocytes, due to their low RNA content, the Seurat object was created from the raw count matrices including all cells with at least 100 genes and 100 UMIs. The percentage of detected mitochondrial genes in *P. berghei* (Pb) and *M. musculus* (Mm) genes and the number of UMI and genes for parasite (PbUMI and PbGenes) and host (MmUMI and MmGenes) were calculated and added as metadata. To exclude duplets, we determined upper thresholds for MmGenes, MmUMI, PbGenes, and PbUMI for each dataset individually by visual inspections of the plots and subsetted datasets on those. Precise thresholds per dataset are listed in table S2. CITE-seq information was added, and barcodes missing in the CITE-seq dataset were excluded. These filtering steps retained 79.20 to 98.53% of all cells per dataset (table S2).

#### Host cell analysis

We analyzed host cells in the spleen and bone marrow by subsetting datasets on cells with more than 100 MmGenes and on features that map to *M. musculus*. Single-cell transcriptomes were normalized using scran (v. 1.14.6) including a clustering step using quickCluster() and computeSumFactors() (*55*). Normalized counts were log +1–transformed. The six datasets from the spleen and bone marrow (two infected and one naïve each) were integrated using the Seurat integration workflow, which is based on identifying anchors across datasets to match shared cell populations (*54*). Cell types were predicted by transferring labels from a bone marrow reference dataset (*24*) using Seurat Transfer Labels workflow and the first 30 principal components.

For individual analysis of spleen and bone marrow cells, the combined object was subsetted per organ to cells containing at least 1000 MmUMI. Following the standard Seurat pipeline, cells were visualized using UMAP nonlinear dimensionality reduction in Seurat with the first 38 and 30 principal components for bone marrow and spleen, respectively. Clusters were identified by a shared nearest neighbor modularity optimization–based clustering algorithm using the FindClusters() function in Seurat using the default parameters and a resolution of 0.6 (bone marrow) or 0.5 (spleen). Conserved markers were identified using the Seurat built-in function with default settings, and clusters were annotated according to marker gene expression, merging similar clusters.

#### Parasite cell analysis

Datasets from the six infected samples (spleen, blood, and bone marrow from two mice) were subsetted on parasite-infected RBCs as defined by (i) having more than 100 PbGenes and (ii) being predicted to be either erythroblasts (based on the label transfer in the host cell analysis) or RBCs (based on having less than 100 MmGenes). We included the MCA dataset as reference (*25*). Normalization with scran and integration of the datasets with Seurat was performed as for the host cell analysis. UMAP nonlinear dimensionality reduction was performed with the first 24 principal components, and clusters were identified using a resolution of 0.51. Clusters were annotated according to correlation with the preannotated stages of the MCA. One cluster with very low UMI count and ambiguous marker genes could not clearly be correlated to any parasite stage and was excluded from further analysis as outlier or duplets.

#### Differential gene expression analysis and gene ontology enrichment

Differential gene expression analysis between *P. berghei* parasites in the spleen, bone marrow, and blood or in normocytes versus reticulocytes was done using edgeR (v. 3.28.1) (*56*, *57*). Single cells were aggregated per cluster, organ, and optionally host cell by summing up counts, and differential gene expression between pseudo-bulk datasets was determined using a quasi-likelihood *F* test. Hits were considered significant based on a false discovery rate smaller than 0.05.

### Statistics

Unless stated otherwise, experiments were repeated at least three independent times, and exact sample sizes are given in the figure legends. Data were analyzed and plotted using GraphPad Prism (v. 9.0.1), except for scRNA-seq data, which were analyzed and plotted in R (v. 3.6.1) using Seurat built-in plotting functions or ggplot2 (v. 3.3.0). Where indicated, data were tested for significant differences using a one-way or two-way analysis of variance (ANOVA), followed by appropriate multiple comparisons tests as indicated in the figure legends. Error bars indicate SEM unless stated otherwise. Significant differences between samples are indicated with asterisks as follows: *P < 0.05; **P < 0.01; ***P < 0.001; ****P < 0.0001.

## Notes

View/request a protocol for this paper from *Bio-protocol*
