# Supplementary material for: Host cell maturation modulates parasite invasion and sexual differentiation in Plasmodium berghei
Source: Sci Adv. 2022 Apr 27;8(17):eabm7348. doi: 10.1126/sciadv.abm7348 (PMC9045723; doi:10.1126/sciadv.abm7348)
Supplement: Supplementary file 1 — Figs. S1 to S9 [file sciadv.abm7348_sm.pdf]

Science Advances
AAAS

Supplementary Materials for

Host cell maturation modulates parasite invasion and sexual differentiation in *Plasmodium berghei*

Franziska Hentzschel, Matthew P. Gibbins, Charalampos Attipa, Dario Beraldi, Christopher A. Moxon, Thomas D. Otto*, Matthias Marti*

*Corresponding author. Email: matthias.marti@glasgow.ac.uk (M.M.); thomasdan.otto@glasgow.ac.uk (T.D.O.)

Published 27 April 2022, *Sci. Adv.* **8**, eabm7348 (2022)
DOI: 10.1126/sciadv.abm7348

The PDF file includes:

Figs. S1 to S9
Legends for tables S1 to S6

Other Supplementary Material for this manuscript includes the following:

Tables S1 to S6

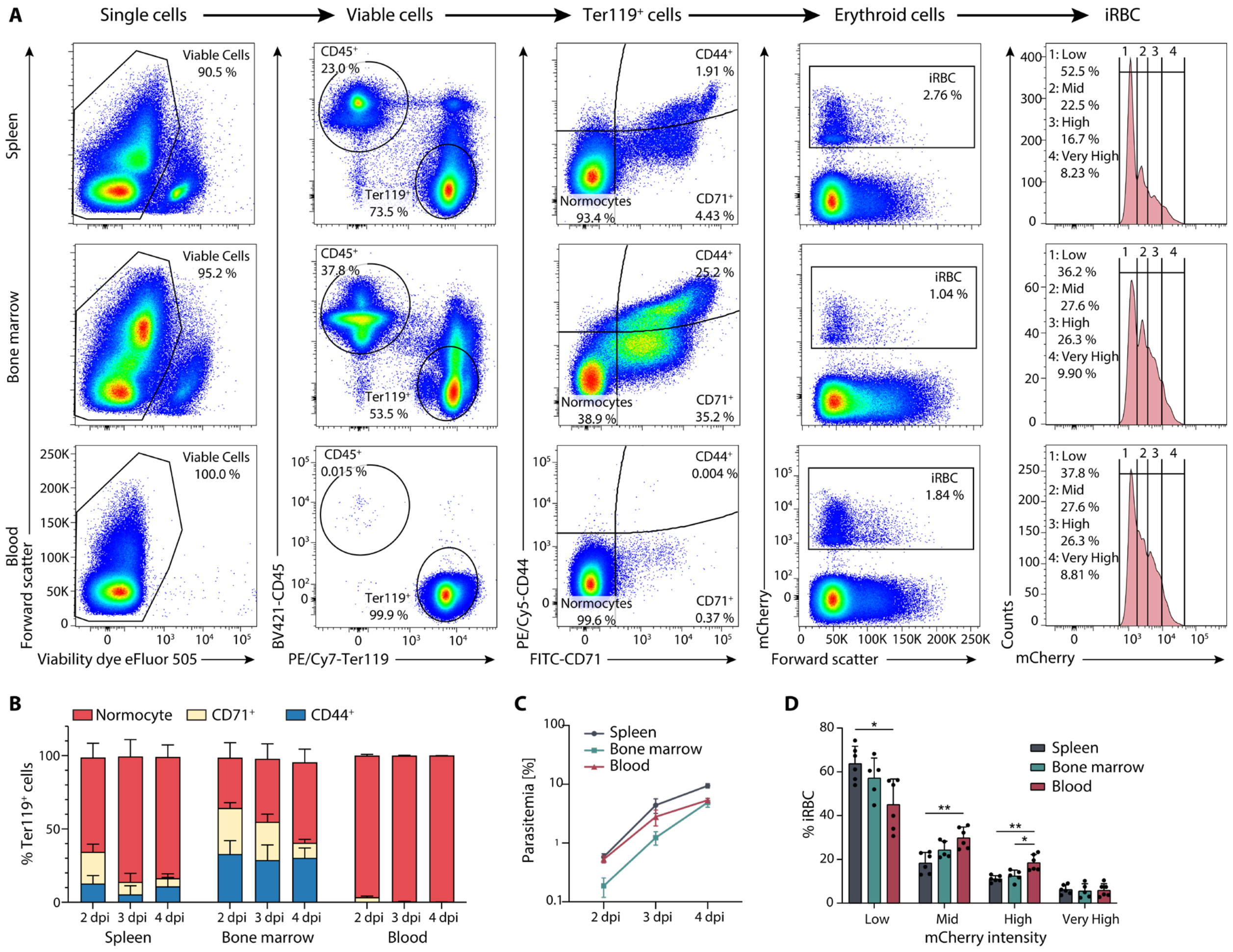

Fig. S1. Time course analysis of *P. berghei* infection in spleen, bone marrow and blood. (**A**) Gating strategy and exemplary flow plots for time course across organs. (**B**) Distribution of host RBCs across organs and time points (n = 6, except for 4 dpi, where n = 3). (**C**) Parasitemia over time in different organs (n = 6, except for 4 dpi, where n = 3). (**D**) Percentage of parasites per mCherry gate per organ as proxy for parasite stages 2 dpi. (n = 6. 2-way ANOVA, Tukey's post test, *, $p < 0.05$, **, $p < 0.01$). B, C, D, mean +/- SEM.

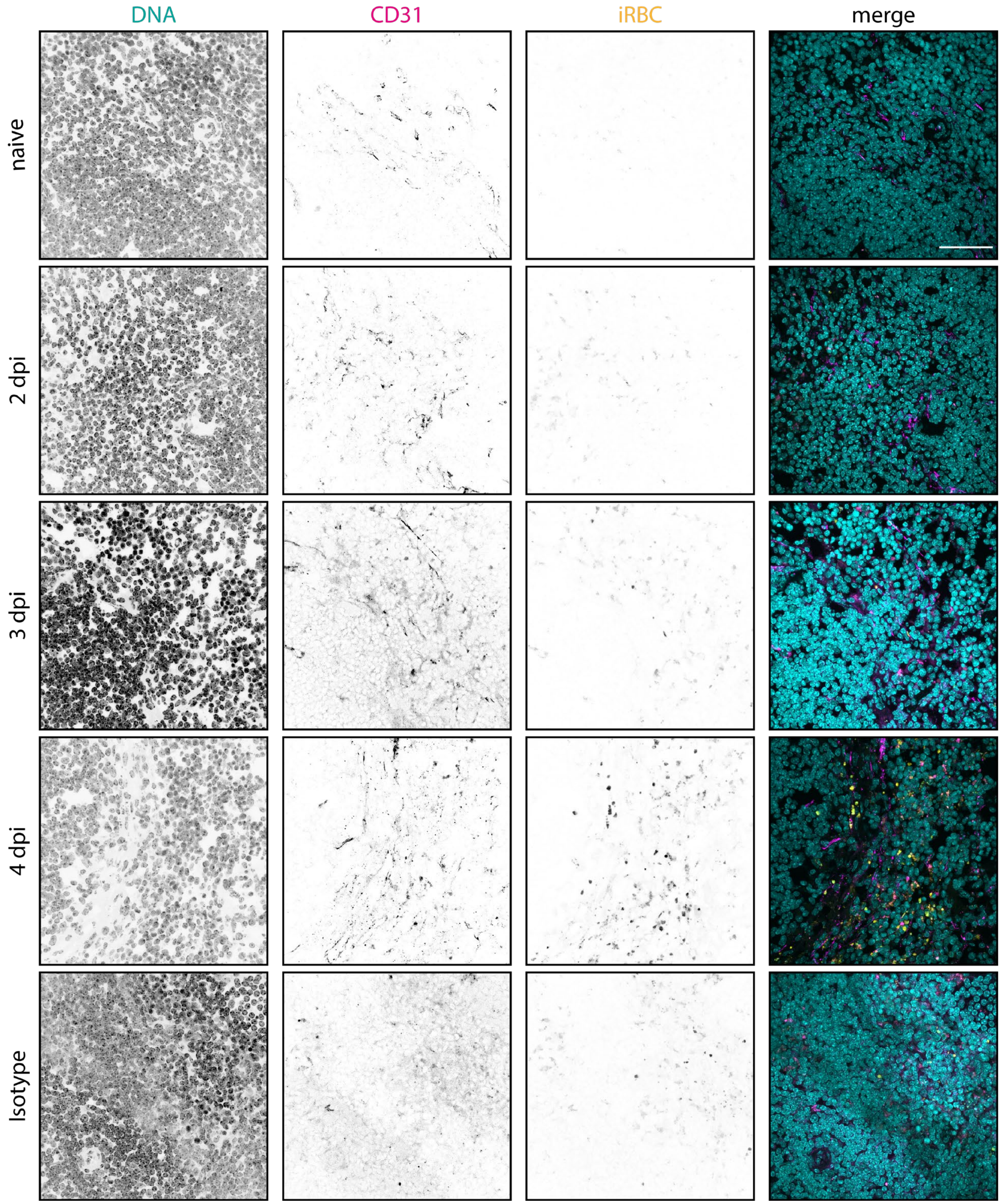

Fig. S2. ***P. berghei*** **localisation in spleen.**

Representative immunofluorescence images of *P. berghei* (pseudocolored in yellow) in spleen sections stained for the endothelial cell marker CD31 (magenta). Bottom row: Isotype control, no CD31 primary antibody. Scale bar 50 µm.

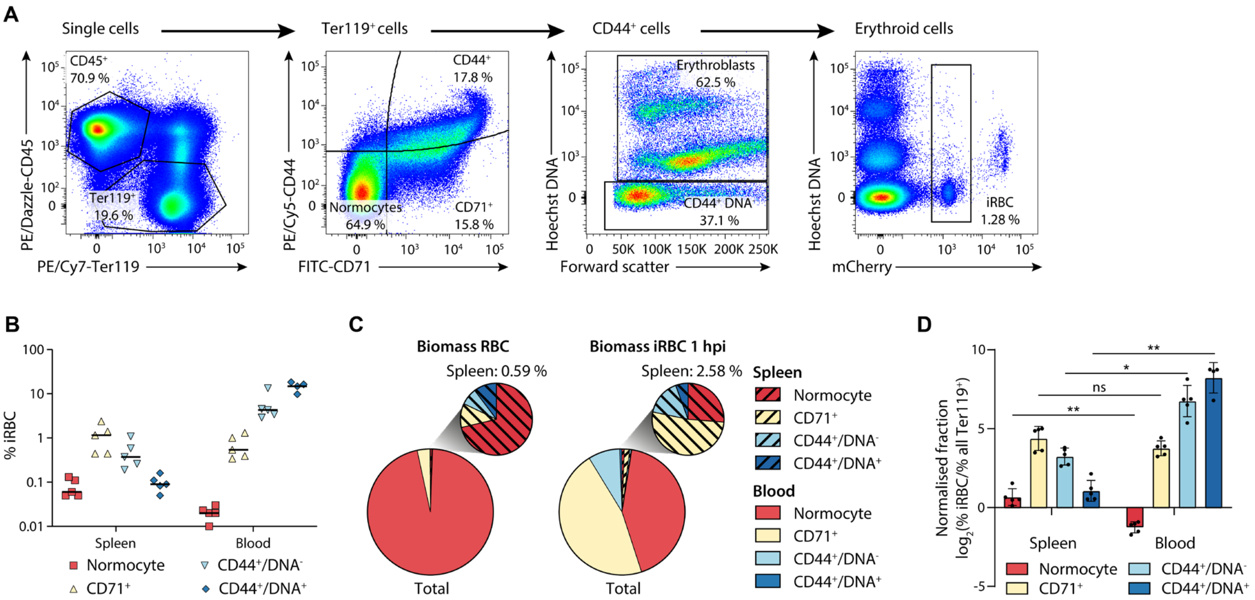

E

Organ	Cell type	High Dose	Low Dose
Spleen	Normocyte	0.56 ± 0.15*	0.66 ± 0.12
	CD71+	1.45 ± 0.27	1.36 ± 0.50
	CD44+/DNA-	0.40 ± 0.09	0.40 ± 0.11
	CD44+/DNA+	0.05 ± 0.01	0.16 ± 0.07
Blood	Normocyte	37.87 ± 2.14	42.49 ± 4.56
	CD71+	56.46 ± 2.13	46.27 ± 4.48
	CD44+/DNA-	3.04 ± 0.50	8.14 ± 0.66
	CD44+/DNA+	0.17 ± 0.06	0.52 ± 0.20

*% of total iRBC

Fig. S3. *P. berghei* invasion preference across organs.

(**A**) Gating strategy to assess ring-stage parasite distribution 1 hpi. (**B**) Parasitemia in splenic and blood RBC cell types one hour post i.v. infection with $4*10^6$ iRBC. (n = 5, line at mean). (**C**) Biomass of RBC cell types (left) and iRBC cell types 1 hpi with $4*10^6$ iRBC (right) in total RBCs (large pie chart) and spleen RBCs (small pie chart) (n = 5). (**D**) Relative enrichment of host cell types in iRBC compared to all RBC 1 hpi with $4*10^6$ iRBC. Values > 0 indicate preferential invasion of this host cell type, values < 0 indicate decreased invasion. (n = 5. 2-way ANOVA, Sidáks post test. mean +/- SEM. ns, not significant, *, $p < 0.05$, **, $p < 0.01$). (**E**) Summary table depicting ring stage biomass ± SEM across organs and host cells 1 hpi with low ($4*10^6$ iRBC) or high ($4*10^7$ iRBC) dose. (n = 5).

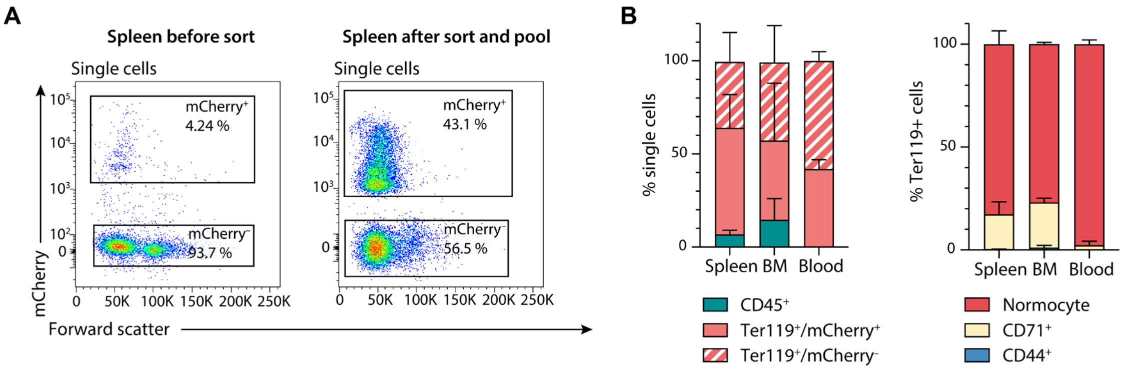

Fig. S4. Flow sorting of iRBCs and host cells prior to scRNA-seq.

(**A**) Exemplary flow plot showing spleen sample and sorting gates before (left panel) and after sorting and 1:1 pooling of mCherry$^+$ iRBC and mCherry$^-$ host cells (right panel). (**B**) Cell type distribution in scRNA-seq samples. (**C**) RBC distribution in Ter119$^+$ cells in scRNA-seq samples. B, C: n = 2, Mean +/- SEM.

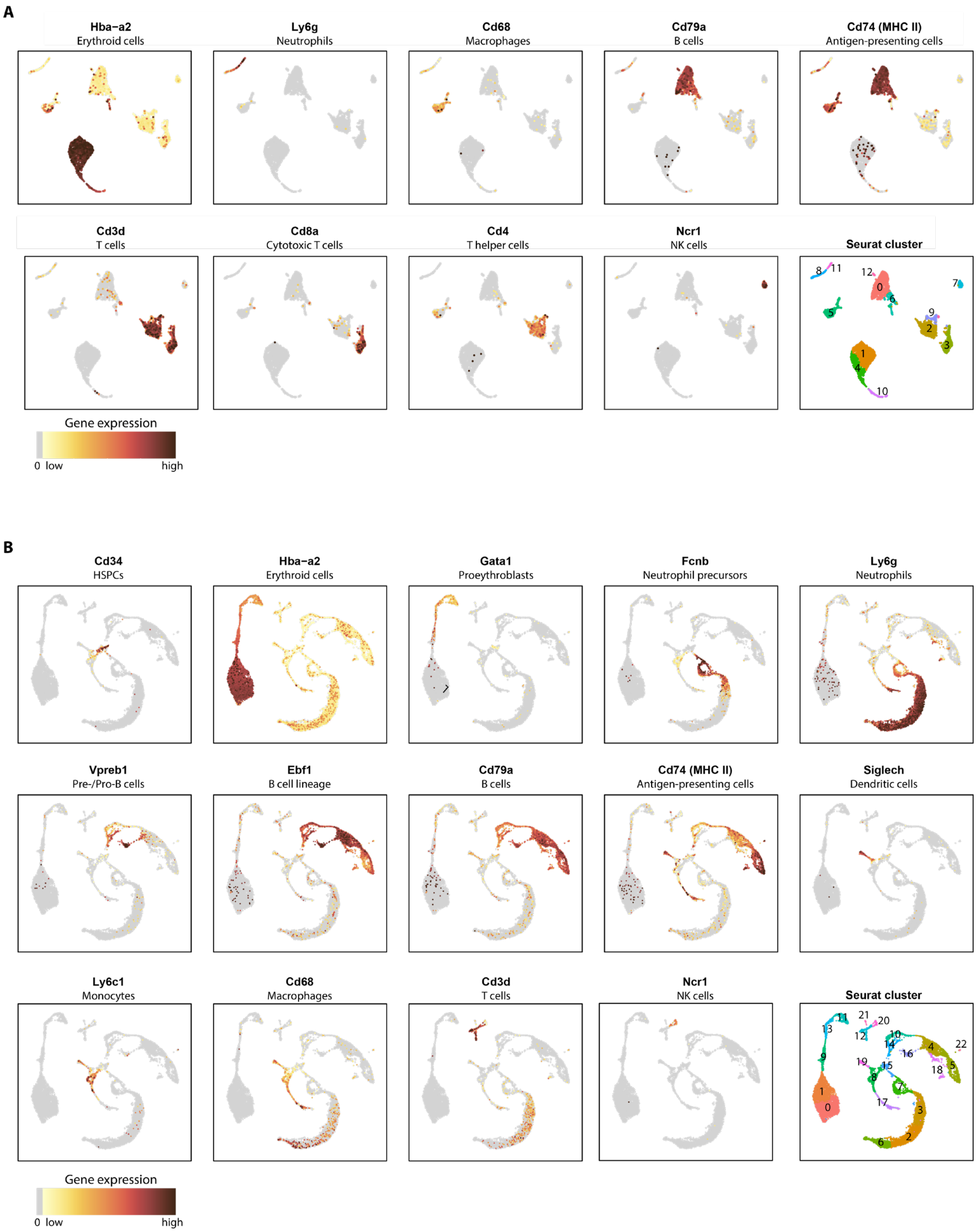

Fig. S5. Marker gene expression in host cells.

(A, B) UMAP of (**A**) spleen and (**B**) bone marrow colored by expression strength of canonical marker genes or according to the original clusters as identified by the unsupervised clustering approach (last panel).

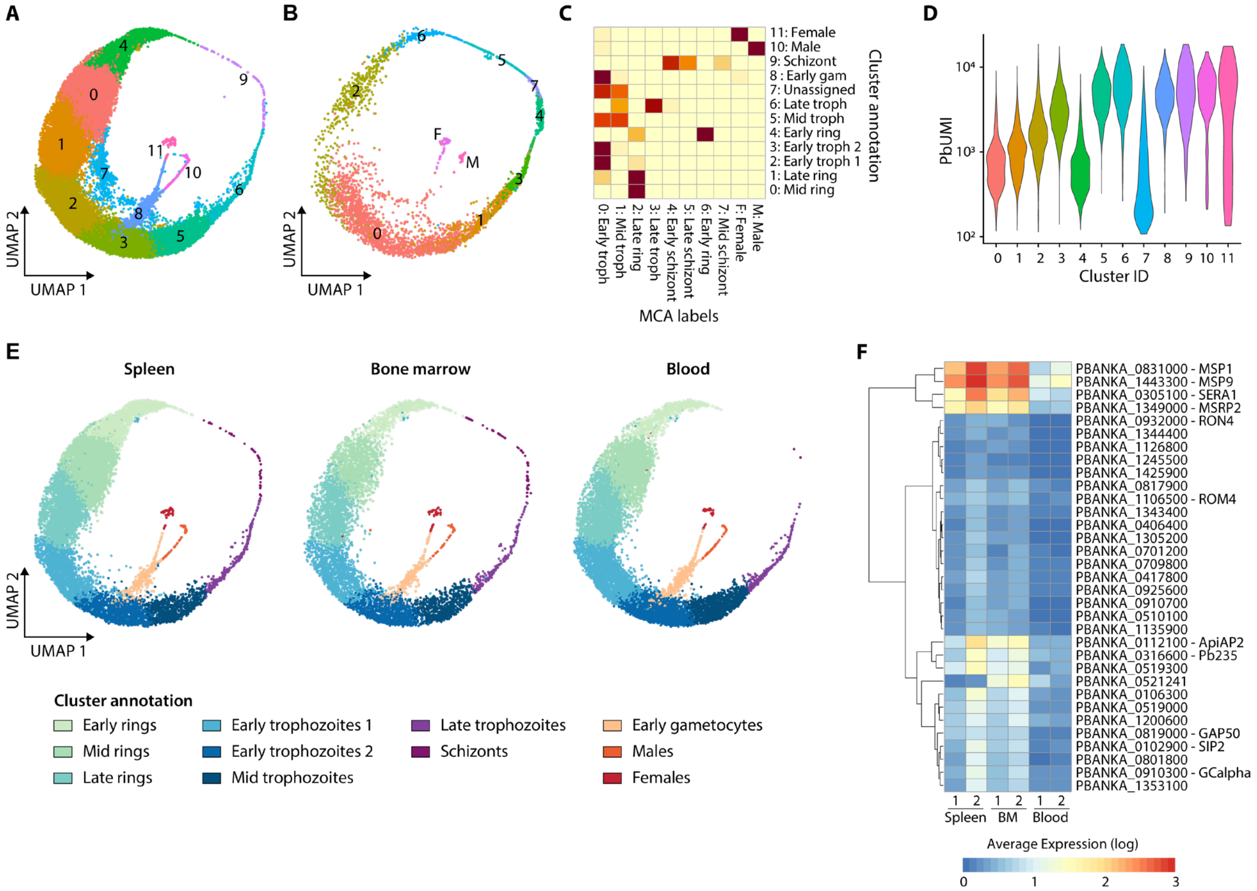

Fig. S6. scRNA-seq analysis of *P. berghei* cells.

(**A**) UMAP of *P. berghei* single cell transcriptomes generated in this study, colored by cluster. (**B**) UMAP of malaria cell atlas. Cells are colored by their annotation in the malaria cell atlas (*15*). The data set was merged with the here generated *P. berghei* data and analysed in parallel. (**C**) Heatmap depicting correlation between MCA annotation and clusters. Clusters were annotated based on the MCA labels they correlated with, and the annotation is indicated next to the cluster number. (**D**) *Pb*UMI per cluster ID. Cluster 7 shows a very low UMI per cell and was excluded from further analysis. (**E**) UMAP of *P. berghei* cells separated according to host organ and colored according to stage. (**F**) Average expression of genes in late trophozoite clusters per sample. Depicted are the most significant hits that are differentially expressed between organs in the late trophozoite cluster. Gene names of annotated genes are listed next to the Gene ID.

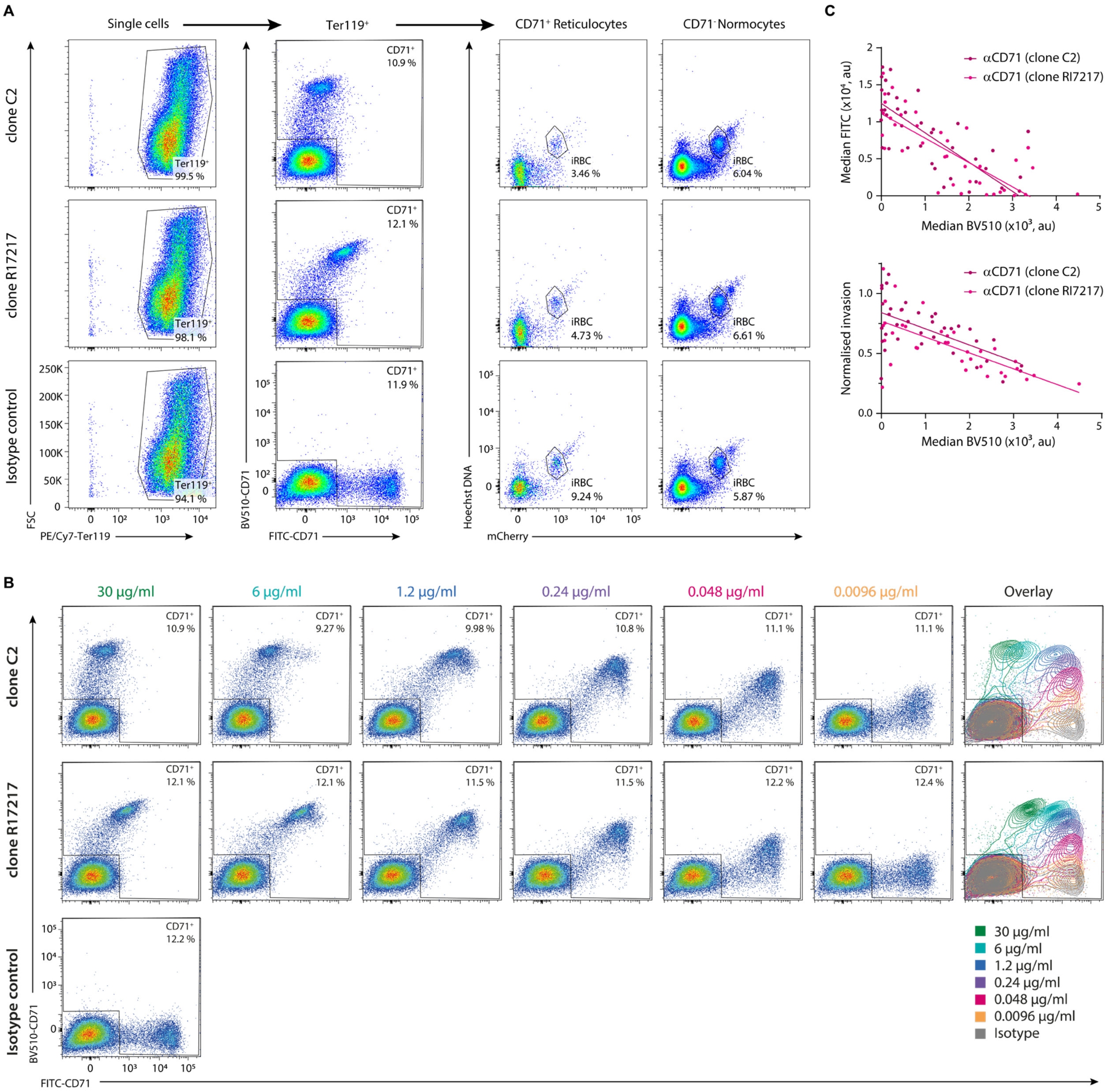

Fig. S7. Flow analysis of CD71-dependent invasion assay.

(**A**) Gating strategy. RBCs were pre-incubated with isotype control or one of two clones of anti-CD71 antibodies (clone C2 or RI7217, both BV510-coupled) before invasion of *P. berghei merozoites*. After invasion, RBCs were co-stained with a second CD71 antibody coupled to FITC and invasion in CD71$^+$ reticulocytes and CD71$^-$ normocytes was determined by flow cytometry. (**B**) Exemplary dot plots showing BV510-CD71 signal (blocking antibody) against FITC-CD71 signal (detection antibody). The binding of the secondary antibody FITC-anti-CD71 decreases with increasing concentration of blocking antibody used. To gate all CD71-positive cells, we chose a gate that includes all BV510$^+$, FITC$^+$ and BV510$^+$/FITC$^+$ events. (**C**) Correlation between median fluorescence of blocking antibody (BV510) and median fluorescence of detection antibody (FITC) (upper panel) or normalised invasion efficacy into reticulocytes (lower panel).

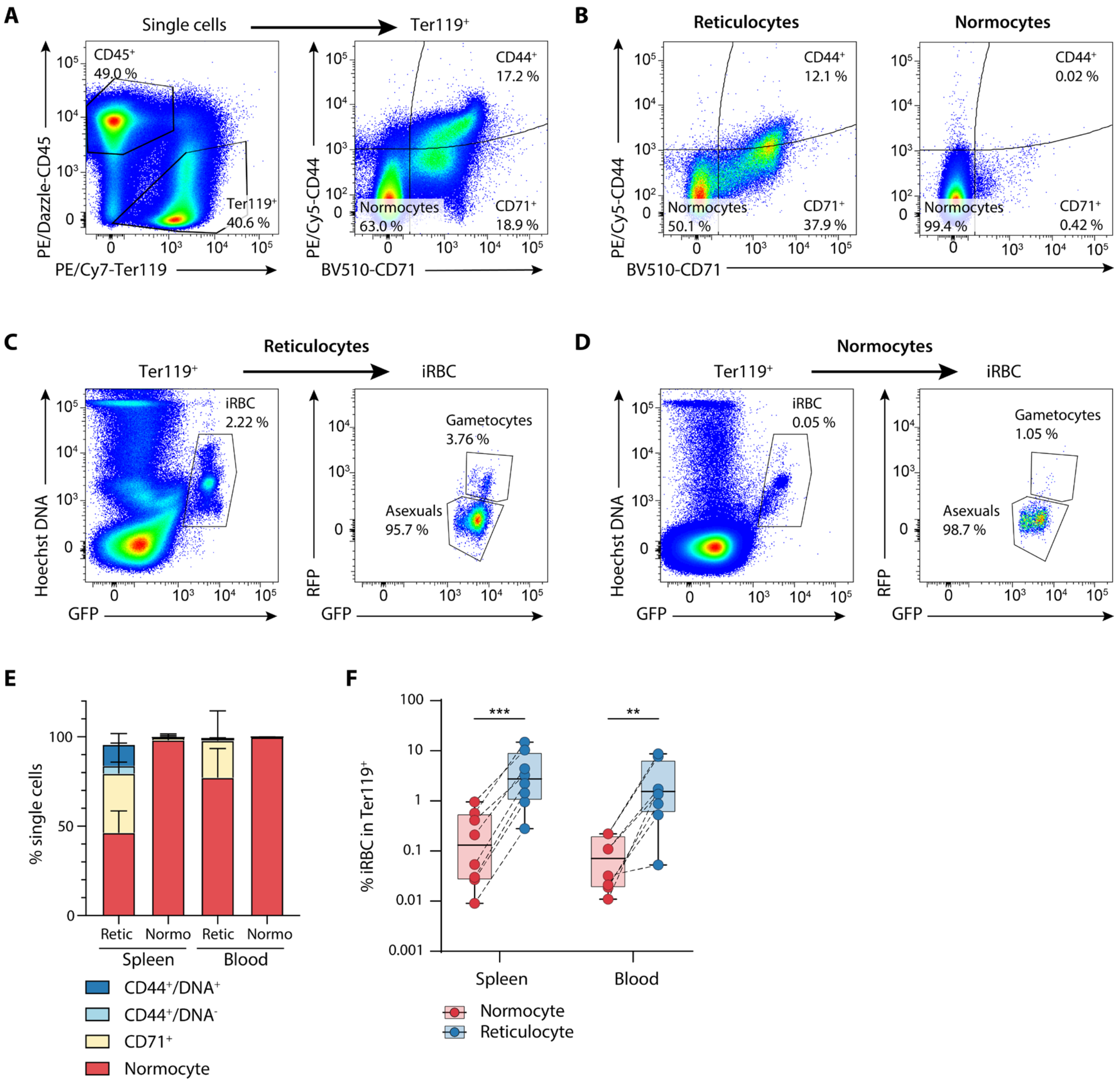

Fig. S8. ***Ex vivo*** **gametocyte conversion in reticulocytes versus normocytes.**

(**A**, **B**) Exemplary flow plot and gating strategy of cells (**A**) before and (**B**) after magnetic cell sorting, yielding a CD71$^+$ enriched reticulocyte fraction and a CD71$^-$ depleted normocyte fraction. (**C**, **D**) Gating strategy for asexual and sexual parasitemia 28 hpi in (**C**) reticulocyte and (**D**) normocyte fractions. (**E**) Host cell distribution in different fractions directly after magnetic cell sorting. (n = 8). (**F**) Parasitemia after 28 h *ex vivo* culture of normocyte- or reticulocyte-enriched iRBC isolated from spleen or blood. Samples from one organ were matched across host cell. (n = 8. Two-way ANOVA, Tukey's post test, **, p < 0.01, ***, p < 0.001).

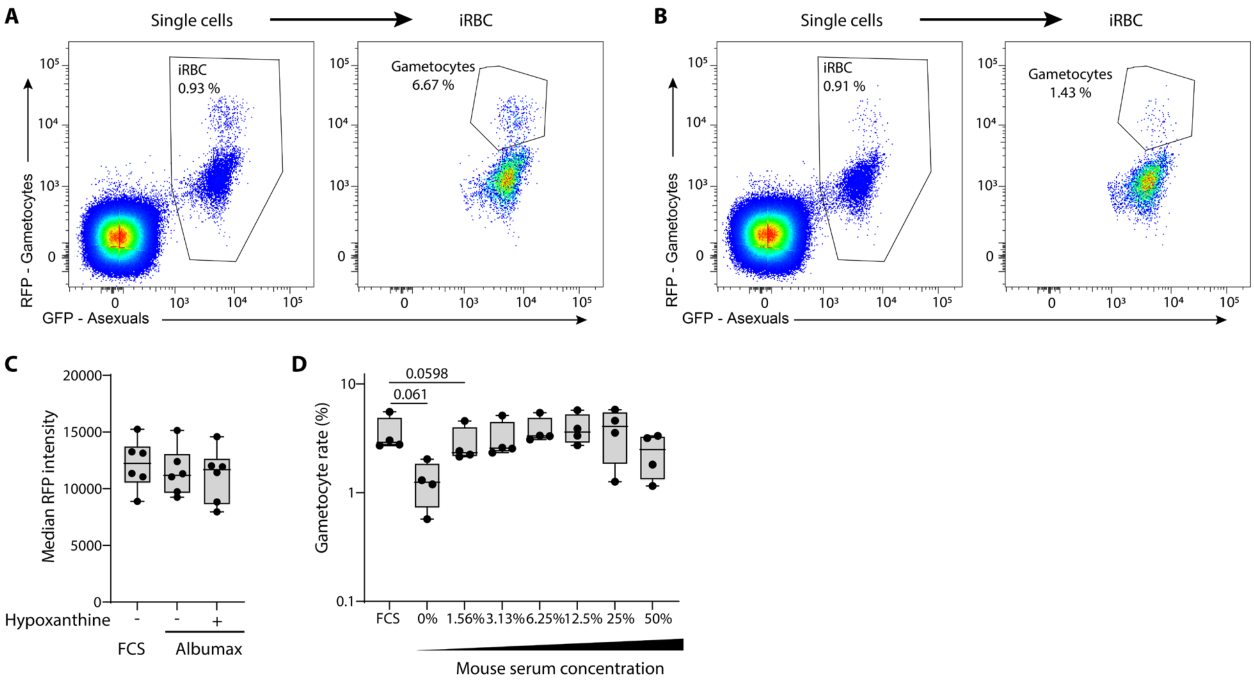

Fig. S9. Nutrient-dependent same-cycle sexual conversion rate.

(A, B) Gating strategy to determine sexual conversion in different media. (**A**) Culture in 20% FCS. (**B**) Culture in 0.5% Albumax/choline. (**C**) Median RFP intensity of gametocytes in FCS/RPMI or Albumax/RPMI. (**D**) Gametocyte rate after *ex vivo* culture in minimal medium supplemented with varying concentrations of mouse serum. (n = 4. One-way ANOVA, Dunnett's post test. relevant p values indicated).

Table S1 (separate file). Histopathological scoring of spleen sections from 3 infected mice.

Table S2 (separate file). Quality control thresholds for filtering scRNAseq data and final cell numbers.

Table S3 (separate file). Marker genes for clusters from bone marrow and spleen single cell analysis.

Table S4 (separate file). Marker genes for clusters from *P. berghei* single cell analysis.

Table S5 (separate file). Differentially expressed genes per cluster between *P. berghei* parasites in different organs.

Table S6 (separate file). Differentially expressed genes per cluster between *P. berghei* parasites in reticulocytes or normocytes.
